# Examining the Impact of Imputation Errors on Fine-Mapping Using DNA Methylation QTL as a Model Trait

**DOI:** 10.1534/genetics.118.301861

**Published:** 2019-04-30

**Authors:** V. Kartik Chundru, Riccardo E. Marioni, James G. D. Prendergast, Costanza L. Vallerga, Tian Lin, Allan J. Beveridge, SGPD Consortium, Jacob Gratten, David A. Hume, Ian J. Deary, Naomi R. Wray, Peter M. Visscher, Allan F. McRae

**Affiliations:** * Institute for Molecular Bioscience, The University of Queensland, Brisbane, Queensland 4072, Australia; † Centre for Cognitive Ageing and Cognitive Epidemiology, Department of Psychology, University of Edinburgh, EH8 9JZ, United Kingdom; ‡ Centre for Genomic and Experimental Medicine, Institute of Genetics and Molecular Medicine, University of Edinburgh, Edinburgh, EH4 2XU, UK; § The Roslin Institute, University of Edinburgh, Midlothian EH25 9RG, United Kingdom; ** Glasgow Polyomics, Wolfson Wohl Cancer Research Centre, University of Glasgow, Bearsden G61 1QH, United Kingdom; †† Mater Research Institute, The University of Queensland, Brisbane, Queensland 4102, Australia; ‡‡ Queensland Brain Institute, The University of Queensland, Brisbane, Queensland 4072, Australia

**Keywords:** fine-mapping, DNA-methylation, imputation, CpG-SNPs

## Abstract

Genetic variants disrupting DNA methylation at CpG dinucleotides (CpG-SNP) provide a set of known causal variants to serve as models to test fine-mapping methodology. We use 1716 CpG-SNPs to test three fine-mapping approaches (Bayesian imputation-based association mapping, Bayesian sparse linear mixed model, and the J-test), assessing the impact of imputation errors and the choice of reference panel by using both whole-genome sequence (WGS), and genotype array data on the same individuals (*n* = 1166). The choice of imputation reference panel had a strong effect on imputation accuracy, with the 1000 Genomes Project Phase 3 (1000G) reference panel (*n* = 2504 from 26 populations) giving a mean nonreference discordance rate between imputed and sequenced genotypes of 3.2% compared to 1.6% when using the Haplotype Reference Consortium (HRC) reference panel (*n* = 32,470 Europeans). These imputation errors had an impact on whether the CpG-SNP was included in the 95% credible set, with a difference of ∼23% and ∼7% between the WGS and the 1000G and HRC imputed datasets, respectively. All of the fine-mapping methods failed to reach the expected 95% coverage of the CpG-SNP. This is attributed to secondary *cis* genetic effects that are unable to be statistically separated from the CpG-SNP, and through a masking mechanism where the effect of the methylation disrupting allele at the CpG-SNP is hidden by the effect of a nearby SNP that has strong linkage disequilibrium with the CpG-SNP. The reduced accuracy in fine-mapping a known causal variant in a low-level biological trait with imputed genetic data has implications for the study of higher-order complex traits and disease.

## Abstract (precis)

This study highlights dangers in over-interpreting fine-mapping results. Chundru *et al.* show that genotype imputation accuracy has a large impact on fine-mapping accuracy. They used DNA methylation at CpG-sites with a variant...

THERE have been a variety of methods proposed for fine-mapping variants discovered in genome-wide association studies (GWAS), with the aim of statistically determining the causal genetic variant, or creating a minimal set of SNPs that contain the causal variant with a high confidence (*e.g.*, Servin and Stephens 2007; Morris 2011; Hormozdiari *et al.* 2014; Kichaev *et al.* 2014; Chen *et al.* 2015; Benner *et al.* 2016; Brown *et al.* 2017; Huang *et al.* 2017). One strong assumption common to all fine-mapping methods is that all possible causal variants are present in the data (Spain and Barrett 2015). This assumption is not satisfied in most studies that use genotypes generated by arrays followed by imputation. While imputation methods with the appropriate choice of reference panel are very accurate for common variants (Mitt *et al.* 2017), imputation errors will still exist and can affect the relative probability of SNPs being determined as causal by fine-mapping methods.

Because of the small number of known causal variants, comparisons of fine-mapping methods need to be performed through simulation, and are often idealized and do not encompass the full range of experimental variation. However, high-throughput measurement of DNA methylation across the genome provides a potential model trait for testing fine-mapping methods. DNA methylation is an epigenetic modification that is influenced by both genetic and environmental factors, with an average heritability of 20% (McRae *et al.* 2014). DNA methylation in humans occurs primarily at CpG dinucleotides, and removal of the CpG sequence through single nucleotide polymorphisms (CpG-SNPs) directly alters DNA methylation at this site (Hellman and Chess 2010; Meaburn *et al.* 2010; Shoemaker *et al.* 2010; Fang *et al.* 2012; Zhi *et al.* 2013). For example, at a CpG locus in a population with a variant with allele frequency of 50% at the C or G, half of the population will have a CpG-site that can be methylated and the other half will not have a CpG site, as the C or G will be substituted with another nucleotide base, and this locus will not be not methylated. Thus DNA methylation at a site with a CpG-SNP provides a trait with a known causal variant of large effect and can be used as a model trait to test fine-mapping. Furthermore, there are large numbers of such sites throughout the genome, and the genetic regulation of methylation by such SNPs have been implicated in disease risk (Dayeh *et al.* 2013; Zhou *et al.* 2015; Chen *et al.* 2016).

In this study, we compare three fine-mapping methods, covering a variety of approaches, Bayesian imputation-based association mapping (BIMBAM) (Servin and Stephens 2007), Bayesian sparse linear mixed model (BSLMM) (Zhou *et al.* 2013), and the J-test (Davidson and MacKinnon 1981), using individual-level SNP data, and DNA methylation at CpG-SNPs as a model trait. We compare 95% credible sets of causal variants for each method, and directly contrast the use of whole-genome sequencing data and imputed genotyping array data, including the choice of imputation reference panel.

## Materials and Methods

### Datasets

#### Lothian birth cohort:

The Lothian birth cohorts of 1921 and 1936 (LBC) (Deary *et al.* 2004, 2007, 2012; Taylor *et al.* 2018) are both part of a longitudinal study on cognitive aging. Participants were all born in 1921 or 1936, and completed a cognitive ability test as part of the Scottish Mental Survey 1932 (Bartlett 1934) or Scottish Mental Survey 1947 (Ensor 1950), respectively. DNA methylation was measured in 1366 study participants using the Illumina HumanMethylation450 BeadChips as described in Shah *et al.* (2014), McRae *et al.* (2018). The mean (SD) age of participants was 79.1 (0.6) from the 1921 cohort, and 69.6 (0.8) from the 1936 cohort. Out of the >400,000 probes remaining after quality control (QC), ∼22,000 have an SNP at the CpG site (CpG-SNP) and a significant methylation QTL (mQTL), with the CpG-SNP being genome-wide significant $\left(P_{CpG‐SNP}<1\times 10^{-10}\right)$ (McRae *et al.* 2018). A set of 1716 sites with a CpG-SNP with a minor allele frequency (MAF) > 0.1 were chosen to make sure we have sufficient power to fine-map the causal variant.

From the LBC, 1370 individuals were whole-genome sequenced on a HiSeq X installation to an average coverage of 36× (minimum 19.6×, maximum 65.9×). All reads were mapped to the build 38 version of the reference genome using BWA (Li and Durbin 2009) and variants called using GATK (DePristo *et al.* 2011) according to its recommended best practices. Variants were annotated using variant effect predictor and gene models from the version 85 release of Ensembl (McLaren *et al.* 2016).

The whole-genome sequence data were compared to array data for the same individuals using PLINK 1.90 (Chang *et al.* 2015). Standard checks for relatedness, heterozygosity, duplication, and sex were also performed. In total, 12 samples were removed from the original 1370 because of failing one or more of these tests. The data were then filtered to include variants that were considered to PASS according to VQSR, had only two alleles, a maximum missingness of 10%, and a minimum genotyping quality of 40.

The imputed datasets were genotyped on the Illumina 610-Quad BeadChip arrays. The data were filtered to remove individuals with high missing rate (>5%), the SNPs with high missing rate (>5%), SNPs with Hardy–Weinberg exact test $P<1\times 10^{-6}$, and SNPs with low MAF (<0.01). We imputed the cleaned data using the 1000 Genomes Project Phase 3 (The 1000 Genomes Project Consortium *et al.* 2015), and the Haplotype Reference Consortium (HRC) reference panels, prephasing the data using EAGLE, and imputing using PBWT on the Sanger imputation server (Durbin 2014; Loh *et al.* 2016; The Haplotype Reference Consortium 2016). The imputed SNPs were filtered again for MAF, deviations from Hardy-Weinberg equilibrium, and a low imputation info score (info<0.8). The chosen info score threshold is quite stringent to prevent low-confidence imputed SNPs having an effect on the fine-mapping analyses. To fairly compare the datasets we use the intersection of the three (*n* = 1166, *m* ≈ 6,300,000 SNPs).

Details of the DNA methylation QC can be found elsewhere (Shah *et al.* 2014; McRae *et al.* 2018). Briefly, DNA methylation was measured on the Infinium HumanMethylation450 array using DNA extracted from whole blood. Raw intensity data were background-corrected and normalized using internal controls, and methylation β values were generated using the R minfi package (Aryee *et al.* 2014). Probes with low detection rate (<95% at *P* < 0.01), and low-quality samples were removed. Individuals with low call rate (<450,000 probes detected at *P* < 0.01) were removed. Probes on the X and Y chromosomes were removed, leaving 450,726 probes remaining. β Values were corrected for BeadChip, sample plate, hybridization date, white blood cell count, and sex.

#### UK10K:

We used the UK10K dataset [European Genome-phenome Archive (EGA) accession numbers: EGAS00001000108 and EGAS00001000090] for the simulations (see Supplemental Material, File S1). The UK10K dataset (UK10K Consortium *et al.* 2015) comprises the whole-genome sequencing of 3781 European individuals from the United Kingdom. The dataset has a total of ∼8,000,000 SNPs after QC (excluding SNPs with Hardy–Weinberg exact test $P<1\times 10^{-6}$, MAF < 0.01, and SNPs with >10% missing data).

#### Systems Genomics of Parkinson’s Disease cohort:

The Systems Genomics of Parkinson’s Disease (SGPD) cohort comprises 956 individuals with Parkinson’s disease, and 930 controls genotyped on the Illumina PsychArray-B.bpm. In our analyses we did not take disease status into account. The data were filtered to remove individuals with high missing rate, the SNPs with high missing rate (>5%), SNPs with Hardy–Weinberg exact test $P<1\times 10^{-5}$, and SNPs with low MAF (<0.01). The imputation was performed using the Sanger imputation server (The Haplotype Reference Consortium 2016) and was imputed using the HRC reference panel (The Haplotype Reference Consortium 2016). The imputed SNPs were filtered again for MAF, deviations from Hardy-Weinberg equilibrium, and a low imputation info score (info<0.3).

The DNA methylation data were measured using the Illumina HumanMethylation450 BeadChip array. Raw intensity data were background-corrected and normalized using internal controls, and methylation β values were generated using the R meffil package (Min *et al.* 2018). Probes of low quality, and low detection rate were removed (<95% at *P* < 0.01). The R meffil package was also used to perform sample QC using Illumina recommended thresholds. Samples were dropped if call rate was low (<450,000 probes detected at *P* < 0.001), if predicted sex (based on XY probes) did not match reported sex, and if predicted median methylated signal was >3 SD from the expected. After these QC steps, methylation β values were quantile-normalized with respect to 20 principal components generated from the control matrix and the most variable probes. Additionally, normalization was adjusted for batch, slide, cohort, sentrix row/column, sex, and age. Of the 1716 probes in the LBC dataset, only 1678 remained after cleaning, thus the replication was only conducted on the respective probes.

##### Simulating phenotypes:

Phenotypes similar to DNA methylation at CpG-SNPs were simulated using the GCTA software (Yang *et al.* 2011). GCTA uses a simple additive genetic model to simulate the phenotypes given the causal variants, with effect sizes drawn form a normal distribution N(0,1). In the case of a single causal variant, the narrow sense heritability is equivalent to the variance explained by the causal variant. We simulated three phenotypes, with $h^{2}=$ 0.2, 0.1, and 0.05, each with 1000 replicates using two sample sizes, the full UK10K dataset (*n* = 3781), and a subset of the UK10K dataset to match the sample size in the imputed LBC dataset (*n* = 1366). The causal variants were chosen at random from the genome, but restricted to have MAF > 0.05.

##### Fine-mapping methods:

To compare the performance of fine-mapping methods, a 95% credible set is constructed for each method, the minimum set of SNPs which will contain the causal SNP 95% of the time. Although the credible set is a Bayesian concept, we can also use a 95% confidence set for Frequentest approach (J-test) because we use the coverage of the causal variant in the sets as a measure of fine-mapping accuracy. Both sets are designed so that 95% of the time the causal variant will be captured. For simplicity we will refer to both sets as credible sets.

The J-test (Davidson and MacKinnon 1981) is a simple regression method to test non-nested hypotheses. The method is as follows:

1. Rank the SNPs by strength of association, and add the most associated SNP to the credible set;
2. Regress the most associated SNP against the phenotype
  $$y=\mu_{1}+X_{1}\beta_{1}+\epsilon_{1};$$
3. Starting at *N* = 2, regress the *N*th best SNP against the phenotype with the fitted values from the regression in step 2 as a covariate:
  $$y=\mu_{N}+X_{N}\beta_{N}+\lambda_{N}\hat{X_{1}}\hat{\beta_{1}}+\epsilon_{N};$$
4. If $\lambda_{N}$ is not significant, we add the SNP to the credible set, increment *N*, and repeat step 3. If $\lambda_{N}$ is significant, we stop here;

where *y* is the phenotype, $X_{i}$ is the genotype of SNP *i*, and $\lambda_{N}$ is the regression coefficient for the fitted values from the regression from step 1. This method tests if the best SNP explains a statistically significant amount of the phenotypic variance more than the *N*th best SNP. To construct a 95% credible set of causal variants, a set of SNPs with 95% probability to contain the causal variant, a Bonferroni-corrected significance of $\frac{P}{N-1}$ was used. To remove redundant tests, only one SNP was tested of SNPs in complete linkage disequilibrium (LD), all SNPs in complete LD that were removed were subsequently added to the credible set if applicable.

BIMBAM (Servin and Stephens 2007), which uses a Bayesian regression approach to find genetic associations, calculates a Bayes factor for each SNP. This is the likelihood of the SNP being causal divided by the likelihood that no SNP in the region is causal. Maller *et al.* (2012) showed that, assuming a single causal variant, the posterior probability of association (PPA) can be written as $PPA_{i}=\frac{BF_{i}}{\sum_{j}BF_{j}}$. This method is used to compute the credible sets, repeatedly taking the next highest associated SNP until a combined posterior probability of association of 95% is reached.

BSLMM (Zhou *et al.* 2013), a mixed-model method, fits SNPs into a mixture of two distributions using a sparsity-inducing prior. BSLMM uses a Markov chain Monte Carlo approach, which is used directly, counting the top associated variant in every 10th iteration, to account for any correlation between iterations. Under the assumption of a single casual variant, the SNP with the largest effect in each iteration is the predicted causal variant. By counting the number of times each SNP is predicted to be the causal variant, the 95% credible set is created by iteratively adding SNPs, in order of most number of counts, until 95% of the total number of iterations is reached $\left(\frac{\sum_{1}^{N}{\text{count}}_{i}}{\sum_{i}{\text{count}}_{i}}\geq 0.95\right)$. In the case of SNPs in complete LD, all SNPs were counted at each iteration.

Many recent fine-mapping methods focus on using summary-level data (Morris 2011; Hormozdiari *et al.* 2014; Chen *et al.* 2015; Benner *et al.* 2016), we attempted to use some of these methods, but FineMap (Benner *et al.* 2016) is unable to handle large effect size traits and CAVIAR (Hormozdiari *et al.* 2014; Chen *et al.* 2015) also ran into computational problems with the large effect size. However, the CAVIAR model is equivalent to the BIMBAM model, as shown in Chen *et al.* (2015), so the comparison is not needed. Other recent fine-mapping methods have focused on integrating functional annotation data to gain extra power (Kichaev *et al.* 2014; Hormozdiari *et al.* 2016), but these functional annotations are highly correlated with DNA methylation so will not be applicable in this case.

##### Conditional analysis:

To check for multiple independent variants affecting the DNA methylation levels two conditional analyses were performed, a conditional and joint (CoJo) method (Yang *et al.* 2012), and a forward selection.

For the forward selection approach, a multiple linear regression can be performed with the top SNP as a covariate,

$$y=\mu +X_{-c}\beta +\sum_{c}X_{c}\lambda +\epsilon ,$$

where *c* is the number of SNPs being conditioned on, *y* is the methylation level, $X_{-c}$ is the N×M−c genotype matrix of all SNPs except the conditioned SNPs, the $X_{c}$ are the N×1 genotype matrices of the SNPs being conditioned on, *μ*, *β*, and *λ* are regression coefficients, and *ϵ* is the error term. If the association is no longer significant $\left(P<5\times 10^{-8}\right)$ when conditioned on the top SNP, then there is only one independent effect, otherwise there are more than one independent variants affecting the DNA methylation in the QTL. We continue to condition on the top SNP from the previous conditional analysis until all the significant associations are removed.

The CoJo model uses a stepwise selection procedure to estimate the number of causal variants. It begins with selecting the most associated SNP, followed by a forward selection step, using a multiple regression conditioning on the chosen SNP. This is followed by a backward selection step by fitting the chosen SNPs in a joint model, and removing any SNPs not significantly associated. The forward and backward selection steps are repeated until no new SNPs are added or removed from the chosen set of SNPs. Between each step the chosen SNPs are checked for multicollinearity (Yang *et al.* 2012).

### Data availability

The LBC methylation data are available at EGA under accession number EGAS00001000910. The LBC1921 and LBC36 genotype data are available on request for relevant research purposes (https://www.lothianbirthcohort.ed.ac.uk/content/collaboration). The UK10K dataset is available from EGA (accession numbers: EGAS00001000108 and EGAS00001000090). The source code used to run the three fine-mapping methods is available on GitHub (https://github.com/chundruv/finemapping_GENETICS2019). Details of the simulation results, the discordance between sequence and genotyped data, and the SGPD consortium member list is provided in File S1. Supplemental material available at Figshare: https://doi.org/10.25386/genetics.7906109.

## Results

### Comparison of fine-mapping approaches

We compare 95% credible sets (the minimum set of SNPs with 95% probability of containing the causal variant) obtained from three fine-mapping approaches using DNA mQTL at a CpG-SNP in the 1166 individuals from the LBC (Deary *et al.* 2004, 2007, 2012; Taylor *et al.* 2018). The performance of the fine-mapping methods is measured by the coverage of the CpG-SNP, which is the proportion of replicates for which the CpG-SNP, the putative causal variant, is present in the 95% credible set. Each fine-mapping approach was applied to both whole-genome sequence data and genotype data from Illumina 610-Quad BeadChip arrays imputed to the 1000 Genomes Project Phase 3 (The 1000 Genomes Project Consortium *et al.* 2015) (LBC-1KG) (*n* = 2504 from 26 populations) and the HRC (The Haplotype Reference Consortium 2016) (LBC-HRC) (*n* = 32,470 Europeans) reference panels (see *Materials and Methods*). Fine-mapping was performed at 1716 DNA methylation sites previously identified to have a *cis*-mQTL $\left(P<1\times 10^{-10}\right)$ in the LBC dataset (McRae *et al.* 2018), with a known common SNP (MAF >0.1) in the CpG site. These DNA methylation sites have a median genetic heritability of 0.86, estimated from a sample of twins and their parents (McRae *et al.* 2014), consistent with a major genetic locus underlying their variation (Figure S1).

**Figure 1:**
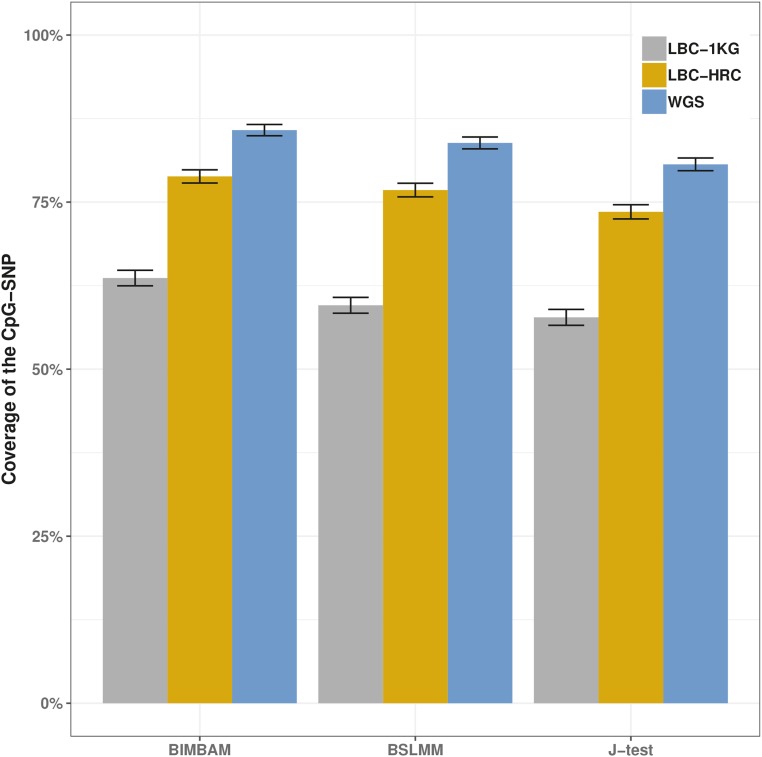
Coverage of the CpG-SNP using three fine-mapping methods. The three methods perform similarly, with only a very small difference in coverage of the CpG-SNP. The coverage of the CpG-SNPs is at a maximum when using whole-genome sequence data, followed closely by the HRC imputed data, with the 1000 Genomes Project imputed data having a much lower coverage of the CpG-SNP.

Under the assumption that the CpG-SNP is causal for the variation in DNA methylation at each site, we measured the performance of the three fine-mapping approaches as the proportion of 95% credible sets of SNPs that included the CpG-SNP (or the method’s coverage), as well as the number of SNPs within each credible set. BIMBAM performed marginally better than both BSLMM and the J-test in terms of coverage of the CpG-SNP, with the trade-off of larger credible sets (Table S1). In the 672 cases where the CpG-SNP was not the most associated SNP (top SNP), the top SNP in the credible sets had a median distance of 2 kb to the CpG-SNP, with 95% of SNPs being within 34 kb. (Figure S2). While performing well on simulated data (see File S1), all three methods failed to reach the expected 95% coverage of the putatively causal CpG-SNP (Figure 1) using either the whole-genome sequence or imputed datasets.

**Figure 2:**
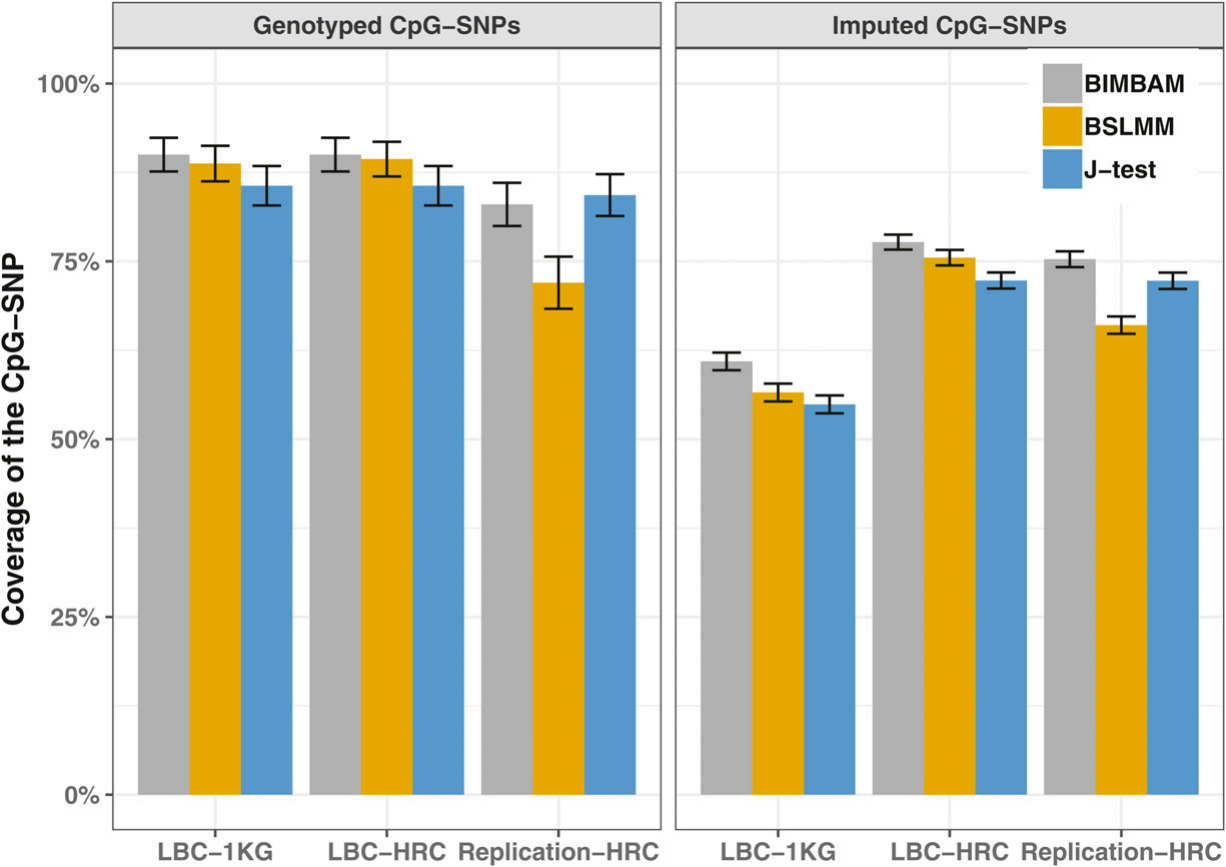
Coverage of the CpG-SNP in those probes where the CpG-SNP is genotyped on the array, and those where it is imputed. The coverage of the CpG-SNP was higher in the probes where the CpG-SNP was genotyped. This result was replicated in an independent dataset imputed using the HRC reference panel (Systems Genomics of Parkinson’s Disease Cohort). When the CpG-SNP is imputed, there is a large difference in the coverage between datasets imputed using the 1000 Genomes Project Phase 3 reference panel (LBC-1KG), and those imputed using the HRC reference panel (LBC-HRC, Replication-HRC).

**Figure 3:**
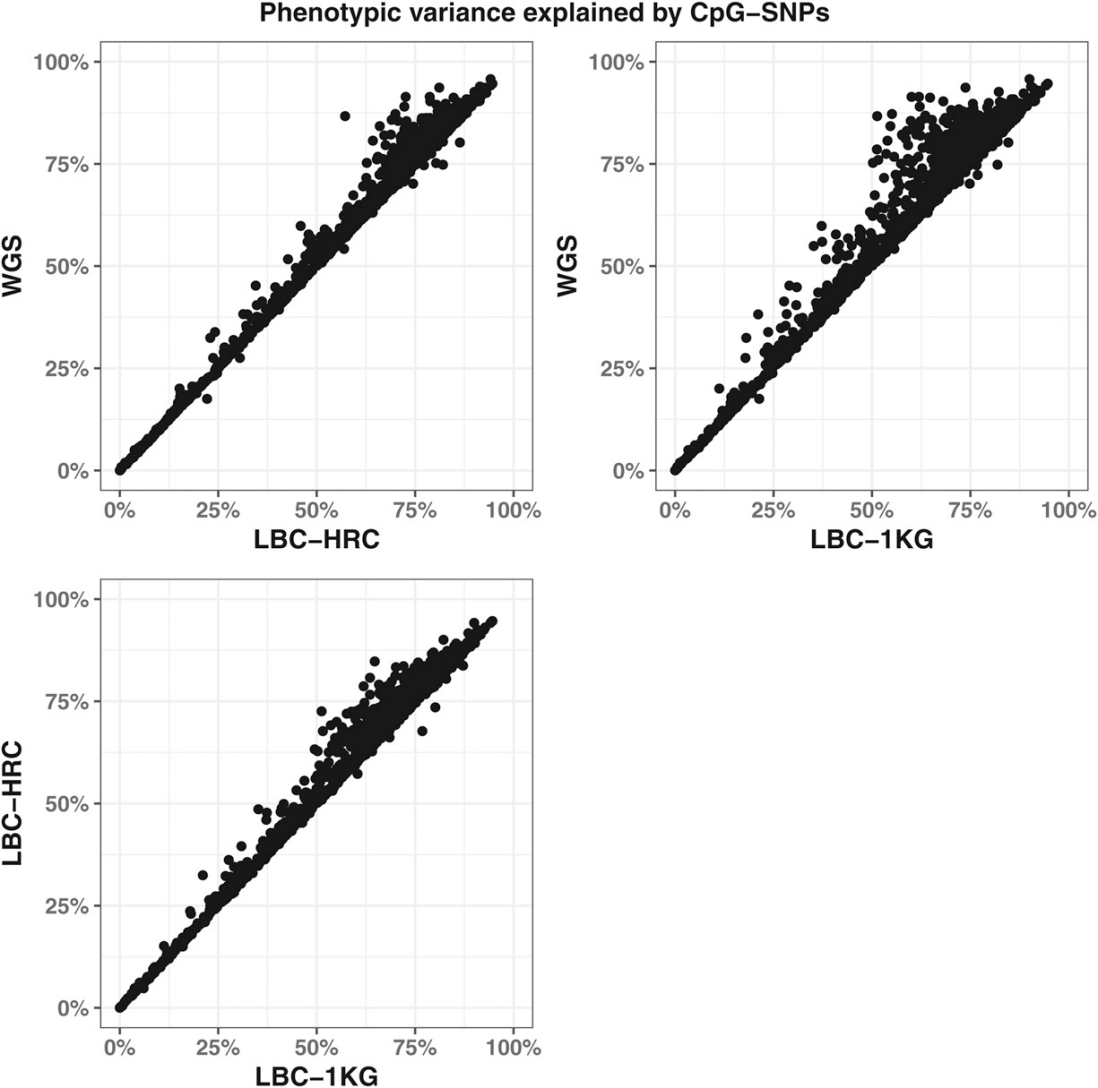
The phenotypic variance explained by the CpG-SNP in the three datasets plotted against one another. Although they are highly correlated, in the top row we observe that the phenotypic variance explained is on average higher in the LBC-WGS dataset than the two imputed datasets, and in the bottom row we observe that the phenotypic variance explained is on average higher in the LBC-HRC dataset than in the LBC-1KG dataset.

Fine-mapping using whole-genome sequence data gave the highest coverage of CpG-SNP, with coverage dropping by ∼7% when comparing to data imputed against the HRC reference and by ∼23% when using the 1000 Genomes Project Phase 3 reference. For the imputed datasets, genotyped CpG-SNPs (160/1716) were included in 95% credible sets between 29 and 33% more often than imputed CpG-SNPs using the 1000 Genomes Project Phase 3 reference, and between 8 and 19% more often using the HRC reference dataset, with this being driven by differences in imputation accuracy (see File S1). The difference between imputed *vs.* genotyped SNPs and overall coverage of 95% credible sets was replicated in an independent dataset of 1886 individuals imputed using the HRC reference panel (Figure 2). The effect of imputation accuracy can also be seen in the phenotypic variance explained by the CpG-SNPs, which is on average higher in the whole-genome sequence dataset than in both the imputed datasets, and the LBC-HRC dataset captures more of the variance than the LBC-1KG dataset (Figure 3).

### Multiple causal variants at DNA methylation cis-QTL

The underlying assumption of our comparison of fine-mapping is the presence of a single causal variant underlying the *cis*-mQTL, with this being implicitly assumed in the construction of the 95% credible set for each of the methods. We performed two analyses to identify mQTL under the influence of multiple genetic variants: a standard forward selection approach and the CoJo stepwise selection model implemented in GCTA-CoJo (Figure S3). Only one independent signal was detected by both methods for 87% of the mQTL. However, when considering only those mQTL showing a single independent association for both methods, we see that the coverage is still below the expected 95% (Table 1).

**Table 1:** The coverage of the CpG-SNP and the size of the credible sets for the probes with a single independent association detected from the both conditional analyses (87% of all probes), using the whole-genome sequence dataset

Method	Coverage (%)	Mean SNPs/set	Median SNPs/set	95% quantile
J-test	82	4	1	14
BIMBAM	87	5	1	19
BSLMM	80	4	1	10

Assuming that the CpG-SNP is the single underlying causal for the DNA methylation levels, we would expect that the CpG-SNP would be captured in at least 95% of the credible sets.

**Figure 4:**
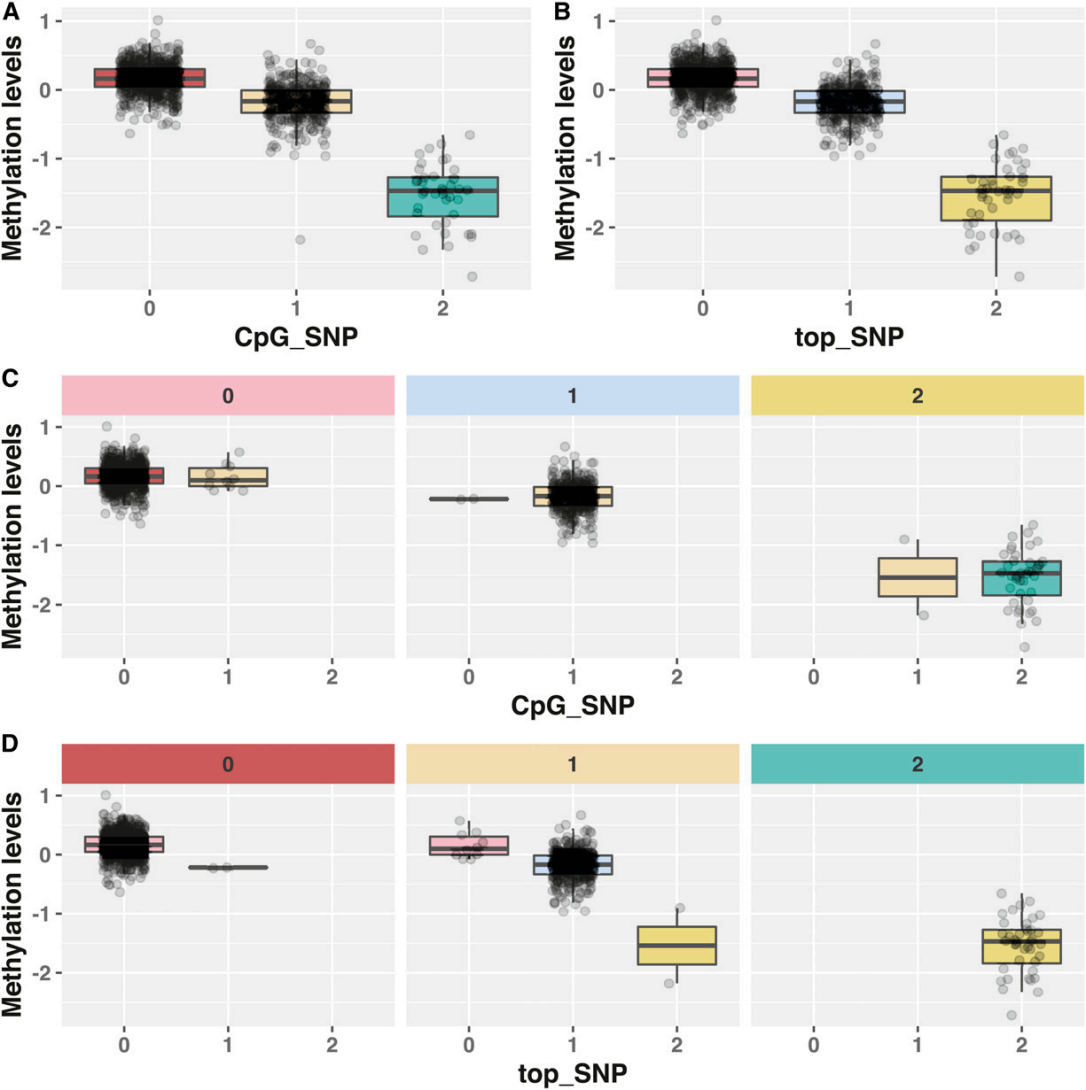
The effect of the CpG-SNP and top SNP on the methylation levels, independent of one another. A and B show the change in methylation levels with a change in the genotype of the CpG-SNP, and the top SNP, respectively, with both having a large effect. C is split into three blocks indicating individuals with 0, 1, or 2 minor alleles at the top SNP, and within each block the points indicate the methylation levels of individuals with 0, 1, or 2 minor alleles at the CpG-SNP, showing there is almost no variation in methylation levels explained by the CpG-SNP after fixing the top SNP. D is the same as the second, except the SNPs are reversed, showing that even after fixing the CpG-SNP there is extra variation in the methylation levels explained by the top SNP.

For the mQTL with one independent association from the conditional analyses, and where the CpG-SNP was not the top SNP, we estimated LD between the top SNP and CpG-SNP. In all cases, the LD between the top SNP and CpG-SNP pairs had a D’ of close to 1, indicating one of the four possible haplotypes between the top SNP and CpG-SNP is not present in our dataset or is very rare. In contrast, the $R^{2}$ measure was highly variable in the cases where the CpG-SNP was not included in the 95% credible set, but close to 1 when it was included (Figure S4). The high D’ and low $R^{2}$ values when the CpG-SNP is not included in the 95% credible interval are consistent with an allele frequency difference between the CpG-SNP and top SNP. In fact, for the cases where the CpG-SNP was not included in the credible set, we observed that one allele of the top SNP captured all the methylation disruption of the CpG-SNP allele as well as several other individuals with low methylation (Figure 4). As such, the top SNP was effectively masking the effect of the CpG-SNP on DNA methylation at these probes.

## Discussion

To capture genuine biological complexity while assessing the performance of fine-mapping methodology, we examined the use of known genetic variation within DNA methylation CpG sites as a model trait. This identified limitations in fine-mapping with imputed sequence data and in statistically separating effects of closely linked variants.

Statistically minimizing the set of potential causal variants underlying the thousands of identified GWAS hits is essential for efficient experimental follow-up. However, we also need to ensure statistically derived sets of potential causal variants actually contain the underlying causal variant. While fine-mapping methods implicitly assume all potential causal variants are available, GWAS generally use imputed genotypes because of large sample size requirements and the relative cost of genotyping arrays *vs.* sequencing. We have shown a dramatic reduction in the proportion of credible sets that actually contain the underlying causal variant when using imputed genotype data, particularly when using the 1000 Genomes Project Phase 3 reference panel for imputation. This imputation panel is still widely used, especially for GWAS meta-analysis combining populations with differing ancestry. In comparison, the more extensive HRC reference panel showed a great reduction in imputed genotype error rates, resulting in increased coverage of the causal variant. This highlights the need to continue the generation of large imputation reference panels across multiple ancestries. The HRC reference panel is ∼6.5 times larger than the African Genome Resource, which is currently by far the largest non-European imputation reference panel.

Although common CpG-SNPs will have a very large effect on the DNA methylation, we were unable to reach the expected 95% coverage of the putatively causal CpG-SNP in our credible sets even when using whole-genome sequenced genotypes. We detected multiple statistically independent genetic associations in the *cis* region surrounding the CpG site for 11% of probes. It is likely that a much higher proportion of probes would be identified as having multiple genetic effects with a greater sample size. In addition, we identified SNPs that effectively masked the effect of the CpG-SNP; these variants had an effect on the methylation levels, and the methylation disrupting allele of these variants were in high LD D’ with the methylation disrupting allele of the CpG-SNP, but at a higher allele frequency, meaning that they masked the effect of the CpG-SNP and explained more of the variance in methylation levels. This is potentially caused by SNPs having a regional effect on DNA methylation; however, arrays do not provide the detailed measures of DNA methylation across a region needed to investigate this further.

The difficulties in fine-mapping a known causal variant in a low-level biological trait have implications for the study of higher-order complex traits and disease. For example, Huang *et al.* (2017) fine-mapped 18 inflammatory bowel disease loci to apparent single-variant resolution. However, their genotype data were based on imputation to the 1000 Genomes Project reference panel, which resulted in >36% of the credible sets in our study not containing the causal variant when compared to whole-genome sequencing. The role of imputation error in the accuracy of fine-mapping also has implications for rarer causal variants. The imputation accuracy for rare variants is much lower than common variants (Mitt *et al.* 2017), implying fine-mapping of rare causal variants will be less accurate than their common counterparts. In addition, fine-mapping approaches that integrate additional epigenetic annotations need to be treated with care. While we could not use such approaches in our study (due to the circular nature of the analysis if applied to mapping DNA mQTL), our results demonstrate that our knowledge of which genetic variants disrupt these epigenetic marks is incomplete. These limitations in statistical fine-mapping need to be recognized when designing functional experiments.
